# Recent advances in m6A RNA modification in hepatocellular carcinoma: from mechanisms to therapeutic potential

**DOI:** 10.3389/fmolb.2026.1772310

**Published:** 2026-03-20

**Authors:** Qingmiao Shi, Yingru Liu, Mengjuan Xuan, Leiya Fu, Na Lou, Chi Lv, Chen Xue

**Affiliations:** 1 Department of Infectious Diseases, The First Affiliated Hospital, College of Clinical Medicine, Henan University of Science and Technology, Luoyang, China; 2 Department of Infectious Diseases, The First Affiliated Hospital of Zhengzhou University, Zhengzhou, China; 3 State Key Laboratory for Diagnosis and Treatment of Infectious Diseases, National Clinical Research Center for Infectious Diseases, National Medical Center for Infectious Diseases, Collaborative Innovation Center for Diagnosis and Treatment of Infectious Diseases, The First Affiliated Hospital, Zhejiang University School of Medicine, Hangzhou, China

**Keywords:** gene expression regulation, hepatocellular carcinoma, molecular targeted therapy, N6-methyladenosine, RNA methylation

## Abstract

Hepatocellular carcinoma (HCC) remains a major global health burden due to its high incidence and mortality, underscoring the urgent need to elucidate the molecular mechanisms of HCC and to improve diagnosis and therapeutic approaches. As the most prevalent form of mRNA modification, N6-methyladenosine (m6A) plays a central role in regulating diverse cellular processes in both physiological and pathological conditions, particularly in cancer initiation, development, and progression. This comprehensive review systematically outlines the biological function of three primary classes of m6A regulator proteins, “writers”, “erasers”, and “readers”. It further examines the dysregulated expression patterns of m6A regulators in HCC, analyzes their clinical associations with tumor grade, clinical stage, and survival outcomes, and evaluates their functional contributions to key oncogenic processes, including cell proliferation, apoptosis, and metastasis. Moreover, this review highlights the critical involvement of m6A RNA modification in orchestrating major molecular mechanisms in HCC, offering an in-depth analysis of its regulatory effects on glycolysis, lipid metabolism, ferroptosis, cancer stemness, tumor immunity, cell cycle progression, and resistance to therapy. Finally, the review synthesizes current progress in emerging therapeutic strategies that target the m6A RNA modification for the treatment of HCC. In conclusion, this review presents a systematic summary of recent advances in m6A RNA modification in HCC, delivering valuable insights for future basic and translational studies. Ongoing research into m6A-mediated regulatory mechanisms hold promise for transforming diagnostic paradigms and enabling the development of innovative therapeutic strategies for patients with HCC.

## 1 Introduction

With a low 5-year survival rate and poor prognosis, hepatocellular carcinoma (HCC) is known as a highly lethal malignancy (Wu et al., 2021). Global cancer statistics from 185 countries in 2020 ranked primary liver cancer as the sixth most common diagnosis and the third greatest contributor to cancer mortality globally, with the HCC subtype representing 75%–85% of cases (Sung et al., 2021). The increasing mortality rates of HCC worldwide are an indication of the urgency for a better understanding of HCC pathogenesis and consequently treatment options (Singal et al., 2023a). Multiple risk factors contribute to the development of HCC, ranging from chronic hepatitis B and C infections to sustained alcohol consumption and chemical-induced liver injury (Llovet et al., 2021). The treatment options available for curative intent for HCC patients include liver transplantation, surgical resection, and local ablative therapies (Singal et al., 2023b). Given the asymptomatic characteristics of HCC, it is frequently diagnosed at advanced stages of the disease. As a result, fewer than 30% of HCC patients are suitable for the above mentioned three radical treatments (Wang and Deng, 2023). Systemic therapy is currently the first-line treatment of HCC (Su G. L. et al., 2022). However, drug resistance is a significant issue affecting HCC patients. There are currently many treatment options for HCC, but they are not sufficient for the treatment of HCC. Therefore, it is imperative to understand the underlying pathways that drive the onset and progress of HCC as this could lead to the discovery of more potent therapeutic approaches for HCC.

Research on post-transcriptional modification of RNAs in eukaryotes is currently gaining momentum. By modifying RNA transcripts, post-transcriptional regulators determine the fate and translation of RNA, ultimately shaping downstream cellular pathways. RNA methylation is a type of post-transcriptional modification. In particular, the N6-methyladenosine (m6A), 5-methylcytosine (m5C), and N1-methyladenosine (m1A) are well-studied RNA methylations that play important developmental roles such as stem cell fate determination and embryonic development (Song et al., 2021). Amongst the different RNA methylations, m6A is the most prevalent and acts on mRNA, and non-coding RNAs such as circular RNA (circRNA) and long non-coding RNA (lncRNA) (An and Duan, 2022). Methyltransferases, demethylases, and RNA-binding proteins regulate m6A modification and are, respectively, known as “writer”, “eraser”, and “reader”. The favorable coordination of these regulators dictates the reversibility and functions of m6A methylation.

Mounting evidence shows that m6A modulates different biological processes involved in the initiation and development of cancers (Ma et al., 2019). Evidence indicates that m6A plays a context-dependent dual role in cancer, with the capacity to either drive or inhibit tumorigenesis, metastasis, and angiogenesis in malignancies such as breast cancer (Zheng et al., 2021). Besides, m6A modification plays pivotal roles in the therapy and prognosis of lung cancer (Diao et al., 2023). Additionally, m6A dysregulation is implicated in the development of gastrointestinal cancers like HCC (Hu et al., 2019). Consequently, research into its role in HCC pathogenesis, treatment, and prognosis has expanded significantly, leading to several insightful reviews. However, as the field matures from mechanistic discovery toward therapeutic application, a distinct need has emerged for a synthesis that not only consolidates the complex regulatory networks of m6A but also critically evaluates the translation of this knowledge into clinical strategies.

This review aims to address this gap by providing a systematic and integrated analysis that explicitly connects the multifaceted roles of m6A in HCC, spanning metabolism, stemness, immunity, cell cycle, and therapy resistance, with the evolving landscape of targeted intervention. Moving beyond foundational mechanisms, we place particular emphasis on dissecting emerging therapeutic modalities, including small-molecule inhibitors and advanced nano-delivery systems, and on discussing the practical pathways and persistent challenges in clinical translation. By adopting this “mechanism-to-therapy” perspective, this work is designed to serve as a forward-looking resource that bridges basic biology with translational oncology, supporting the development of novel strategies to overcome treatment resistance and improve outcomes for patients with HCC.

## 2 Regulators of m6A RNA modification

The regulators of m6A RNA modification can be broadly classified as “writer”, “eraser” and “reader”. In the m6A modification process, “writer” complexes catalyze the addition of methyl groups to RNA, while “eraser” enzymes facilitate their removal. The modified sites are then recognized by “reader” proteins, which directly mediate downstream effects on RNA stability, translation, and function.

**FIGURE 1:**
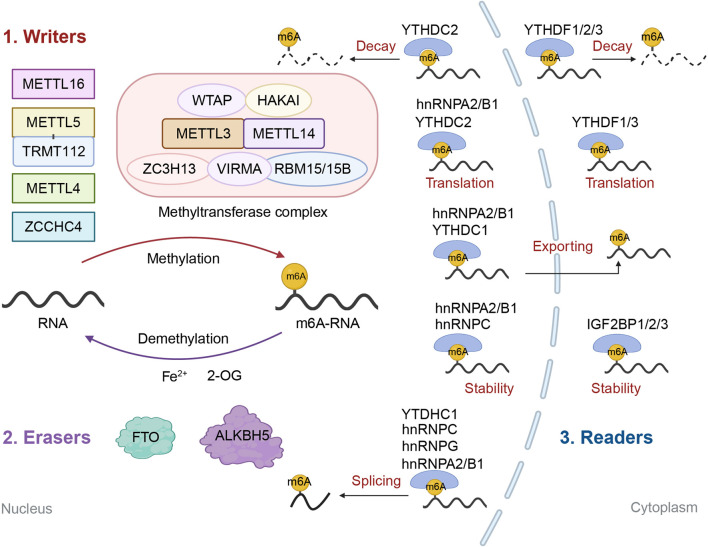
Regulators of m6A RNA modification. The m6A regulators can be divided into three categories, namely “writers”, “erasers” and “readers”. “Writers” and “erasers” are responsible for the reversible regulation of RNA m6A modification by respectively catalyzing methylation and demethylation of RNAs. m6A “writers” include methyltransferase complex, which is composed of a stable heterodimer (METTL3-METTL14) and some regulatory subunits (WTAP, HAKAI, ZC3H13, VIRMA, RBM15/15B). Besides, there are some m6A “writers” (METTL16, METTL4, METTL5–TRMT112 complex, ZCCHC4) functioning independently. FTO and ALKBH5 are two main m6A “erasers”. The m6A “readers” are responsible for identifying m6A and exerting the functions of m6A, including decay (YTHDF1/2/3 and YTHDC2), translation (hnRNPA2/B1, YTHDC2 and YTHDF1/3), exporting (hnRNPA2/B1 and YTHDC1), stability (hnRNPA2/B1, hnRNPC and IGF2BP1/2/3) and splicing (YTHDC1, hnRNPC, hnRNPG and hnRNPA2/B1) of RNAs.

According to consensus motif analyses, the m6A modification sites, described as the DRACH motif (D = A, G or U; R = G or A; H = A, C, or U; where A is converted to m6A; A, G, C, U are four kinds of RNA nucleotides) are located mainly in the coding sequences (CDS), 3′-untranslated regions (3′-UTRs), and particularly around the stop codon. This genomic distribution was first revealed by the seminal transcriptome-wide mapping studies (Dominissini et al., 2012; Meyer et al., 2012). The following sections will elaborate on the m6A regulators and their functions in the modulation of m6A modification (Figure 1).

### 2.1 “Writers”

Methyltransferase complex (MTC) is one of the most important m6A writers. It is made up of catalytic subunit m6A-METTL complex (MAC) and the regulatory subunit m6A-METTL-associated complex (MACOM) (Su S. et al., 2022). MAC is a functional heterodimeric complex composed of methyltransferase-like 3 (METTL3) and methyltransferase-like 14 (METTL14), which is critical for installing m6A modifications on nuclear RNAs (Lence et al., 2019; Liu et al., 2014). Additionally, several studies have shown that METTL14 is not only an RNA-binding platform but is also required for substrate recognition (Wang et al., 2016). MACOM includes Williams tumor 1-associated protein (WTAP), as well as the newly discovered subunits. As a member of the regulatory subunit MACOM, WTAP interacts with METTL3 and METTL14 and is essential for the MAC’s methylation activity. It functions by modulating the RNA-binding capacity of METTL3, thereby guiding the recruitment of the MAC to its target mRNAs (Ping et al., 2014). Thanks to the advancement of sequencing technology, other MACOM subunits, such as Vir-like m6A methyltransferase associated (VIRMA, also known as KIAA1429), zinc finger CCCH-type containing 13 (ZC3H13), RNA binding motif protein 15/15B (RBM15/15B) and human *CBLL1* gene encoding E3 ubiquitin-protein ligase, HAKAI, were recently discovered (Bawankar et al., 2021; Wen et al., 2018; Xu et al., 2021). A new study reported that VIRMA likely stabilizes the RNA methylation function of the METTL3-METTL14 heterodimer by sequestering the complex away from double-stranded DNA (dsDNA), thereby ensuring its availability for RNA targets (Yan et al., 2023). Additionally, VIRMA preferentially mediates the methylation of m6A sites located in 3′UTR and close to the stop codon (Yue et al., 2018). The m6A methylation of RNAs requires the perfect coordination of each subunit within the MTC. The MTC catalyzes most of the m6A modifications on mRNAs (Li Y. J. et al., 2024). Other methyltransferases mediate the m6A modifications on non-coding RNAs and some mRNAs. METTL16 is an m6A methyltransferase, which mainly exerts its RNA methylation function in the nucleus (Su R. et al., 2022). It can bind with the U6 small nuclear RNA (snRNA) and MAT2A mRNA to initiate their m6A modifications (Satterwhite and Mansfield, 2022). METTL5 forms a stable complex with tRNA methyltransferase subunit 11-2 (TRMT112), an interaction essential for its stability (Van Tran et al., 2019). This METTL5–TRMT112 complex principally mediates the m6A modification of 18S ribosomal RNA (rRNA) (Turkalj and Vissers, 2022). The m6A modification of 28S rRNA, on the other hand, is catalyzed by zinc finger CCHC-containing domain 4 (ZCCHC4) (Van Tran et al., 2019). METTL4 is necessary for the U2 snRNA n6,2′-O-dimethyladenosine (m6Am) methylation (Goh et al., 2020).

### 2.2 “Erasers

In 2011, Jia et al. reported the demethylation activity of fat mass and obesity-associated protein (FTO) on the m6A-modified nuclear mRNA and confirmed that m6A-modified nuclear mRNA is the physiological substrate of FTO (Jia et al., 2011). Therefore, FTO became the first widely recognized m6A demethylase. Dysregulation of FTO is implicated in multiple disease states, with cancer being a prominent example (Li Y. et al., 2022). In addition to FTO, alk B homolog 5 (ALKBH5), is an m6A-specific demethylase (Huang et al., 2020; Zheng et al., 2013). FTO and ALKBH5 are also nonheme Fe(II) and 2-oxoglutarate (2-OG)-dependent dioxygenases and require nonheme Fe(II) and 2-OG for the demethylation process (Kuschman et al., 2023). The discovery of m6A demethylases shows the m6A modification process is dynamically regulated.

### 2.3 “Readers”

There are several types of m6A readers, including the YT521-B homology domain-containing family (YTHDF) proteins, YTH domain containing (YTHDC) family, insulin-like growth factor 2 mRNA-binding protein (IGF2BP) family, and heterogeneous nuclear ribonucleoprotein (hnRNP) family. These m6A readers directly bind to and determine the fate of m6A-modified RNAs (Wang et al., 2015). The YTHDF family includes three functionally distinct homologs, YTHDF1, YTHDF2, and YTHDF3, located in the cytoplasm (Zaccara and Jaffrey, 2020). YTHDF1 and YTHDF3 modulate the degradation and translation of m6A-modified mRNAs (Li J. et al., 2022; Liu et al., 2020; Shi et al., 2017). YTHDF2, on the other hand, drives mRNA decay through its recruitment of the CCR4-NOT complex, which is formally known as the carbon catabolite repression 4 - negative on TATA-less complex (Du et al., 2016). YTHDC1 and YTHDC2 are members of the YTHDC family and mainly exert their functions in the nucleus (Chen X. et al., 2022). YTHDC1 orchestrates mRNA splicing by recruiting precursor messenger RNA (pre-mRNA) splicing factors and modulating their access to the binding regions on target mRNAs (Xiao et al., 2016). It also regulates the nuclear export of mRNAs (Qiao et al., 2023). Some studies show an association between YTHDC2 and the translation and degradation of mRNAs (Ma et al., 2023; Yuan et al., 2022). However, one study reported that the function of YTHDC2 in gametogenesis has nothing to do with the m6A modification (Saito et al., 2022). Therefore, the native function of YTHDC2 on RNAs needs to be further studied. The IGF2BP family, consisting of IGF2BP1, IGF2BP2, and IGF2BP3, possesses the K homology domains essential for recognizing mRNA. This family targets the consensus GG(m6A)C sequence and affects the stability of the m6A-modified mRNAs (Huang et al., 2018). The hnRNPA2/B1, hnRNPC, and hnRNPG, some of the few homologs in the hnRNP family, are m6A readers. hnRNPA2/B1 modulates diverse RNA processes, including transcription, splicing, transport, stability, and translation (Liu and Shi, 2021). hnRNPC relates to the stability and splicing of mRNA (Huang et al., 2021; Zhu et al., 2022). hnRNPG regulates the alternative splicing of m6A-modified pre-mRNA (Zhou et al., 2019). The Arg-Gly-Gly repeats in the C-terminal domain mediate its binding to m6A-modified RNA motifs (Liu et al., 2017).

## 3 Clinical significance of aberrant m6A RNA modifications in HCC

In recent years, accumulating research has demonstrated that m6A regulator dysregulation is critically involved in the pathogenesis and progression of HCC, and the level of m6A regulators is closely associated with the tumor progression, clinicopathological characteristics and prognosis of patients (Xu C. et al., 2025). This section will systematically expound the expression patterns, clinical associations and multifunctional biological roles of various m6A regulators in HCC (Table 1), aiming to outline a complete association map of the m6A regulatory network and the clinical outcomes of HCC.

**TABLE 1:** Clinical significance of aberrant m6A RNA modifications in HCC.

Types	m6A regulators	Expression (tumor vs. normal)	Prognosis	Clinical significance	Function role	Ref.
Eraser, Reader	FTO, YTHDF2	Upregulation	Poor	Tumor volume and weight, immune evasion	Promoting proliferation, migration and invasion of HCC cells	Xu et al. (2025a)
Writer	METTL3	Upregulation	Poor	OS,clinical stage and lymph node metastasis, tumorigenesis and lung metastasis	Promoting HCC cell proliferation, invasion, migration, immune process, CD8+T cell apoptosis and repressing HCC cell apoptosis	Xu et al. (2025b)
Writer	METTL3	Upregulation	Poor	OS, disease-specific survival, histological grade, pathological stage, AFP level	Promoting proliferation and inhibiting apoptosis of HCC cells	Wang et al. (2025c)
Writer	WTAP	Upregulation	Poor	Median survival time	Overexpressing WTAP in CD8^+^ T cells significantly facilitated cell proliferation, invasion, and colony formation, while inhibiting apoptosis in HCC cells	Li et al. (2025c)
Writer	WTAP	Upregulation	Poor	IHC score, HCC differentiation, OS	Creating an immune-suppressive microenvironment	Sun et al. (2025b)
Writer	METTL4, METTL5	Upregulation	Poor	OS, RFS, HCC recurrence	—	Zhao et al. (2025a)
Writer	METTL5	Upregulation	Poor	OS, disease-free survival	—	Ye et al. (2023)
Writer	METTL14	Downregulation	Poor	—	Leading to oxidative stress and cell death of HCCcells	He et al. (2025)
Writer	METTL14	Downregulation	Poor	—	Overexpression of METTL14 Suppresses the migration and invasion of HCC cells	Rong and Jiang (2025)
Writer	ZC3H13	Downregulation	Poor	OS, RFS	Promoting immune infiltration in HCC	Wu et al. (2022a)
Eraser	FTO	Downregulation	Poor	OS, RFS	Promoting proliferation, migration and invasion of HCC cells	Zhou et al. (2025)
Eraser	FTO	Upregulation	Poor	TMN stage, metastasis, OS	Promoting proliferation, invasion, and EMT in HCC cells	Zhang et al. (2025b)
Eraser	ALKBH5	Upregulation	Poor	OS	Promoting proliferation, colony formation, migration, invasion and anchorage-independent malignant growth of HCC cells	Wang et al. (2025a)
Eraser	ALKBH5	Downregulation	Poor	OS, progression-free survival, tumor weight, liver metastasis nodules	Promoting proliferation, migration and colony formation and decreasing apoptosis of HCC cells	Ma et al. (2025)
Reader	YTHDF1	Upregulation	Poor	OS, Pathology stage	—	Zhao et al. (2018)
Reader	YTHDF2	Upregulation	Poor	OS	—	Wang et al. (2024d)
Reader	YTHDF3	Upregulation	Poor	Cancer recurrence	Promoting proliferation, migration and invasion of HCC cells	Chen et al. (2024b)
Reader	IGF2BP2	Upregulation	Poor	Tumor volume and weight	Promoting proliferation, migration and invasion of HCC cells	Zhao et al. (2025c)
Reader	IGF2BP3	Upregulation	Poor	OS and disease-free survival	Promoting proliferation, migration, and invasion of HCC cells	Du et al. (2025)
Reader	IGF2BP3	Upregulation	Poor	OS, tumor volume and weight	Promoting proliferation, migration and colony formation of HCC cells	Zhao et al. (2025b)

### 3.1 “Writers”: breaking the balance between promoting and inhibiting cancer

During the generation stage of m6A modification, the functions of different components within the “writer” complex exhibit astonishing diversity, and the disruption of their balance is a key event driving the malignant progression of HCC. Among them, METTL3 is the most thoroughly studied and well-supported cancer-promoting factor. A large amount of clinical data consistently indicates that METTL3 is significantly upregulated in HCC tissues (Chen W. et al., 2025; Liu et al., 2024). Elevated METTL3 expression correlated significantly with reduced overall survival (OS) and recurrence-free survival (RFS) in patients, and was closely linked to malignant clinical features such as later clinical stage, lymph node metastasis, higher histological grade and alpha-fetoprotein (AFP) level (Li D. et al., 2025; Xu W. et al., 2025). Functioning as a potent oncogene, METTL3 drives HCC tumor growth by enhancing tumor cell proliferation, invasion, migration, and colony formation, while concurrently suppressing apoptosis (Peng et al., 2025; Sun T. et al., 2025; Wang Y. et al., 2025). Animal experiments further verified the key role of METTL3 in promoting tumorigenesis and lung metastasis of HCC (Xu W. et al., 2025). It is worth noting that recent studies have also revealed new functions of METTL3 in the tumor immune microenvironment, such as its ability to participate in inducing apoptosis of CD8^+^ T cells, suggesting that it has extensive regulatory capabilities beyond tumor cells themselves (Xu W. et al., 2025).

As a core component of the MACOM, WTAP exhibits clear cancer-promoting properties as well. Its elevated expression in HCC is linked to poorer median survival time, overall survival, and even worse degree of tumor differentiation in patients (Li R. et al., 2025). Functionally, WTAP drives HCC progression through promoting tumor cell proliferation, invasion and clone formation and facilitating immune evasion by exhaustion of CD8^+^ T cells. A remarkable study highlights its unique mechanism of driving tumors by shaping an immunosuppressive microenvironment (Sun Z. et al., 2025). In addition, METTL4 and METTL5 upregulation has been observed in HCC, predicting poor OS, RFS and a higher risk of recurrence, jointly supporting the cancer-promoting effect of m6A writers in HCC (Ye et al., 2023; Zhao J. et al., 2025).

However, not all m6A “writers” serve as HCC promoters. METTL14 and ZC3H13 mainly play the role of tumor suppressor factors in HCC. In contrast to METTL3, METTL14 frequently shows downregulated expression in HCC, and its low expression state predict unfavorable patient prognosis (Rong and Jiang, 2025). Functionally, downregulation of METTL14 leads to oxidative stress and cell death, thereby promoting tumor progression (He et al., 2025). On the contrary, upregulation of METTL14 can effectively inhibit the migration and invasion abilities of HCC cells and then exert anti-cancer effects (Rong and Jiang, 2025). Similarly, the downregulation of ZC3H13 also indicates poor OS and RFS, and it is believed to exert a tumor suppressor effect by promoting the infiltration of anti-tumor immune cells in tumor tissues (Wu S. et al., 2022). These findings reveal the antagonism and balance of the internal functions of the m6A “writer”, which have a decisive impact on the ultimate outcome of HCC.

### 3.2 “Erasers”: background-dependent complex regulator

The m6A “eraser” mediates the removal of methylation modifications from RNA. Among them, the roles of FTO and ALKBH5 in HCC demonstrate a high degree of complexity and background dependence. Research on FTO has revealed its perplexing dual role. One study has reported that reduced FTO expression in HCC correlates with poor OS and RFS, and drives malignant progression by promoting proliferation, migration and invasion of HCC cells (Zhou et al., 2025). However, another study has reached the opposite conclusion, finding that FTO upregulation drives tumor aggressiveness by stimulating proliferation, invasion, and EMT, thereby linking its high expression to advanced TNM stage, metastasis, and poor overall survival (Zhang L. et al., 2025). Similarly, ALKBH5 also has contradictory reports in HCC. Existing studies suggest that its upregulation can promote the proliferation, colony formation, migration, invasion and anchorage-independent malignant growth of HCC cells to promoting HCC development (Wang K. T. et al., 2025). Studies indicate that downregulation of ALKBH5 enhances the proliferation, migration, and colony formation of HCC cells while suppressing apoptosis. Consequently, its reduced expression is associated with advanced disease features such as poorer OS, progression-free survival, intrahepatic metastasis and greater tumor burden (Ma et al., 2025).

This seemingly contradictory result is likely to stem from the high heterogeneity of HCC itself, different etiological backgrounds (such as HBV, HCV, non-alcoholic steatohepatitis, etc.) and differences in the tumor microenvironment. It indicates that FTO and ALKBH5 act as precise molecular regulators, with their ultimate effects highly dependent on the specific molecular context and cellular state.

### 3.3 “Readers”: a consistent executor of malignant phenotypes

Unlike the complexity of “erasers”, the role of m6A “readers” in HCC is particularly consistent and clear. As the terminal executors of the m6A regulatory network, they strongly promote the malignant phenotype of HCC. Members of the YTHDF family (YTHDF1, YTHDF2, YTHDF3) and the IGF2BP family (IGF2BP2, IGF2BP3) show almost consistent upregulated expression in HCC and have been widely confirmed as independent risk factors for poor prognosis (Chen S. et al., 2024; Du et al., 2025; Wang H. et al., 2024; Zhao L. et al., 2025; Zhao et al., 2018; Zhao Y. et al., 2025). Upregulation of YTHDF1 is related to later pathological stages and poor OS (Zhao et al., 2018). YTHDF2 is also associated with shortened OS and is related to tumor immune microenvironment (Wang H. et al., 2024). The upregulation of YTHDF3 is closely linked to a higher risk of tumor recurrence (Chen S. et al., 2024). Functionally, YTHDF3 promotes the proliferation, migration and invasion of HCC cells to promote HCC progression. Similarly, IGF2BP2 and IGF2BP3 have also been confirmed to be powerful cancer-promoting factors. IGF2BP2 induces tumor growth of HCC in mice by promoting proliferation, migration and invasion of HCC cells (Huang et al., 2025). High expression of IGF2BP3 promotes proliferation, migration, invasion and colony formation of HCC cells and significantly correlates with shorter OS, disease-free survival in patients and larger tumor volume and weight in mice (Du et al., 2025; Zhao L. et al., 2025).

### 3.4 The antagonistic dynamics and functional competition among m6A readers

The functional outcome of m6A modification is not determined by a single m6A reader in isolation, but is frequently governed by the competition and antagonism between different readers for common target transcripts. This dynamic interplay constitutes a critical layer of post-transcriptional regulation. In cancer, the balance between mRNA stabilization and degradation directly influences oncogene expression levels and cellular fate. In HCC, a pivotal competitive axis exists between readers that promote mRNA decay and those that enhance mRNA stability.

Strong evidence for this functional antagonism comes from the study of AMOTL1 mRNA in HCC. While YTHDF2 and IGF2BP2 bind to distinct m6A sites on the same AMOTL1 transcript, they exert opposing effects. IGF2BP2 stabilizes the mRNA and promotes cell proliferation, whereas YTHDF2 binding accelerates its degradation and suppresses proliferation. The ultimate cellular phenotype depends on which reader dominates, establishing a clear paradigm of functional competition for a shared oncogenic mRNA target (Zhang et al., 2026). Furthermore, direct competition for an identical m6A site may also occur. For instance, E2F3 mRNA in HCC has been reported as a substrate where YTHDF2 and IGF2BP2 competitively bind to the same m6A residue. Their binding equilibrium is modulated by a regulatory circular RNA, thereby influencing E2F3 expression and tumor growth (Chen Z. et al., 2022). This provides a preliminary model for site-specific reader competition. The foundational observation that different readers, such as YTHDF1 and YTHDF2, can target overlapping sites of mRNAs also underscores the potential for such competitive interactions (Wang et al., 2015).

It is important to note that rigorous evidence demonstrating direct, physical competition between distinct reader proteins for the exact same m6A nucleotide to dictate the fate of a defined oncogene in HCC remains limited. This represents an active area of investigation. The dynamic balance of this competition is likely fine-tuned by upstream signals, cellular context, and intermediary factors, highlighting a critical frontier in understanding the complexity of the m6A regulatory network and its therapeutic implications.

## 4 Regulatory mechanisms of m6A RNA modification in HCC

The m6A RNA modification plays a crucial role in the pathogenesis and development of HCC. In this section, we aim to discuss the roles of m6A RNA modification in several biological processes known to be altered in cancers, such as glycolysis, lipid metabolism, ferroptosis, cancer stemness, tumor immunity, cell cycle progression, and therapy resistance (Table 2).

**TABLE 2:** Regulatory mechanisms of m6A RNA modification in HCC.

Types	m6A regulators	Function	Related RNA	Involved biological process	Mechanism	Ref.
Writer	METTL14	Suppressor	USP48 mRNA	Aerobic glycolysis	METTL14→USP48↑→SIRT6 stabilization↑→attenuating glycolysis	Du et al. (2021)
Writer	METTL3	Oncogene	HIF-1α mRNA	Aerobic glycolysis	HBXIP→METTL3↑→M6A modification of HIF-1α↑→promoting aerobic glycolysis	Fan et al. (2021)
Writer	ZC3H13	Suppressor	PKM2 mRNA	Aerobic glycolysis	ZC3H13→ the stabilization of PKM2 mRNA↓→Aerobic Glycolysis↓	Wang et al. (2021)
Writer	METTL3	Oncogene	lncRNA SLC2A1-DT	Aerobic glycolysis	c-Myc→METTL3↑→YTHDF1-lncRNA SLC2A1-DT stabilization↑→β-catenin→c-Myc→promoting glycolysis	Zeng et al. (2024)
Eraser	FTO	Oncogene	GLUT1, PKM2, c-Myc mRNA	Aerobic glycolysis	FTO-IT1→FTO↑→GLUT1,PKM2,c-Myc↑→promoting glycolysis	Wang et al. (2023a)
Reader	YTHDF3	Oncogene	PFKL mRNA	Aerobic glycolysis	YTHDF3 →PFKL↑→promoting aerobic glycolysis	Zhou et al. (2022)
Reader	IGF2BP2	Oncogene	CDC45 mRNA	Aerobic glycolysis	IGF2BP2→the stabilization of CDC45 mRNA ↑→promoting glycolysis	Wu et al. (2023)
Reader	IGF2BP2	Oncogene	HK2, SLC2A1 (GLUT1) mRNA	Aerobic glycolysis	miR4458HG-IGF2BP2→t HK2 and SLC2A1(GLUT1) mRNA↑→promoting glycolysis	Ye et al. (2023)
Writer, reader	METTL3, YTHDC1	Oncogene	LINC00294	Aerobic glycolysis	METTL3/YTHDC1→the stabilization of LINC00294↑→HK2 and GLUT1↑→promoting aerobic glycolysis	Zhang et al. (2023)
Writer	METTL3/14	Oncogene	ACLY, SCD1 mRNA	Lipid metabolism	METTL3/14 →ACLY and SCD1↑→promoting triglyceride and cholesterol production and accumulation of lipid droplets	Yang et al. (2022)
Writer	METTL3	Oncogene	SCAP mRNA	Lipid metabolism	METTL3→SCAP mRNA↑→activating cholesterol biosynthesis	Pan et al. (2023)
Writer	METTL3	Oncogene	LINC00958	Lipid metabolism	METTL3→LINC00958↑→sponged miR-3619-5p→HDGF↑→promoting lipogenesis	Zuo et al. (2020)
Writer	METTL5-TRMT112	Oncogene	18S rRNA	Lipid metabolism	METTL5-TRMT112→m6A-modified 18S rRNA→promoting fatty acid metabolism	Peng et al. (2022)
Eraser	ALKBH5	Oncogene	LINC01468 RNA	Lipid metabolism	ALKBH5→LINC01468 RNA stabilization and upregulation→activiting PI3K/AKT/mTOR signaling pathway→promoting *de novo* lipid biosynthesis	Wang et al. (2022a)
Writer	METTL14	Suppressor	SLC7A11 mRNA	Ferroptosis	Hypoxia→HIF-1α/METTL14/YTHDF2/SLC7A11 axis→inhibiting ferroptosis	Fan et al. (2021)
Writer	WTAP	Oncogene	ATG5 mRNA	Ferroptosis	WTAP/YTHDC2→ATG5↑→promoting ferroptosis	Li et al. (2024b)
Writer	KIAA1429	Oncogene	SLC7A11 mRNA	Ferroptosis	KIAA1429→heightening the activity of SLC7A11→inhibiting ferroptosis	Wang et al. (2023b)
Reader	IGF2BP3	Oncogene	SLC7A11 mRNA	Ferroptosis	LINC00942/IGF2BP3→SLC7A11 mRNA stabilization↑→inhibiting ferroptosis	Jin et al. (2024)
Reader	IGF2BP1	Oncogene	PARK7 mRNA	Ferroptosis	IGF2BP1→PARK7 stability↑→inhibiting ferroptosis	Zhao et al. (2023)
Writer	METTL3	Oncogene	FZD10 mRNA	Cancer stemness	METTL3→FZD10 activity→activating β-catenin and YAP1→promoting the stemness property	Wang et al. (2023c)
Writer	METTL3	Oncogene	lncRNA LINC00106	Cancer stemness	METTL3→LINC00106↑→sponging let7f→ periostin activation→activiting PI3K-AKT signaling pathway→promoting stemness property	Liang et al. (2021)
Writer	METTL3	Oncogene	SOCS3 mRNA	Cancer stemness	METTL3→SOCS3 mRNA stability↓→SOCS3↓→JAK2/STAT3 pathway↑→promoting stemness	Hu et al. (2024)
Eraser	FTO	Oncogene	NANOG, SOX2 and KLF4 mRNA	Cancer stemness	FTO→NANOG, SOX2 and KLF4↑→promoting stemness	Bian et al. (2021b)
Reader	YTHDF2	Oncogene	OCT4 mRNA	Cancer stemness	YTHDF2→OCT4↑→promoting stemness	Zhang et al. (2020)
Reader	YTHDC1	Oncogene	circHPS5	Cancer stemness	YTHDC1 →cytoplasmic output of circHPS5→sponging miR-370→HMGA2 ↑→promoting stemness	Rong et al. (2021)
Reader	IGF2BP1	Oncogene	MGAT5 mRNA	Cancer stemness	IGF2BP1→MGAT5 mRNA stability↑→promoting stemness	Yang et al. (2021)
Reader	IGF2BP1	Oncogene	lncRNA MIR4435-2HG	Cancer stemness	IGF2BP1→MIR4435-2HG↑→protecting NOP58 from degradation→promoting stemness	Zhu et al. (2023)
Writer	WTAP	Oncogene	circCCAR1	Tumor immune	WTAP/IGF2BP3→circCCAR1 stability↑→facilitating CD8 + T-cell dysfunction	Hu et al. (2023)
Reader	YTHDF2	Oncogene	ETV5 mRNA	Tumor immune	YTHDF2→ETV5↑→PD-L1 and VEGFA↑→promoting immune evasion	Wen et al. (2024)
Writer	METTL3/16	Oncogene	lncRNA ZNNT1	Tumor immune	METTL3/16→ZNNT1↑→osteopontin (OPN)↑→recruiting and inducing M2 polarization of macrophages	Wei et al. (2024)
Writer	METTL3	Oncogene	RBM14 mRNA	Tumor immune	METTL3→RBM14↑→promoting M2-phenotype polarization of KCs	Hu et al. (2022)
Reader	YTHDF1	Oncogene	EZH2 mRNA	Tumor immune	YTHDF1→EZH2↑→IL-6↑→mediating MDSC recruitment and activation→CD8 T-cell dysfunction	Wang et al. (2023e)
Writer	WTAP	Oncogene	ETS1 mRNA	Cell cycle	WTAP→EZH2↓→p21 and p27↓; CDC25C, CDK1, cyclin-A2 and cyclin-B1↑→modulating the G2/M phase	Chen et al. (2019)
Writer	METTL3	Oncogene	lncRNA MAPKAPK5_AS1	Cell cycle	METTL3→MAAS↑→c-Myc→activating CDK4, CDK6 and Skp2→facilitating G1/S transition	Tao et al. (2022)
Reader	YTHDF2	Oncogene	MCM2/5 mRNA	Cell cycle	YTHDF2→MCM2 and MCM5 mRNA stability↑→MCM2 and MCM5↑→promoting cell cycle progression	Yang et al. (2023)
Writer	METTL3	Oncogene	SLC7A11 mRNA	Radioresistance	METTL3→IGF2BP2 mediated SLC7A11 mRNA stability↑→SLC7A11 protein↑→Radioresistance; METTL3↑→m6A-YTHDF2→SOCS2 mRNA decay↑→ubiquitination of SLC7A11 protein↓→SLC7A11 protein↑→Radioresistance	Zhang et al. (2025a)
Writer	METTL16	Oncogene	LAMA4 mRNA	Chemoresistance	METTL16→LAMA4 mRNA stability↑→LAMA4↑→COL4A1↑→DDP resistance	Cao and Bi (2025)
Writer	WTAP	Oncogene	FOXM1 mRNA	Chemoresistance	WTAP↓→FOXM1 mRNA↓→DDR↓→cisplatin sensitivity↑	Huang et al. (2025)
Writer	METTL3	Oncogene	TUG1	Chemoresistance	METTL3↓→TUG1↓→miR-9↑→EIF5A2↓→doxorubicin resistance ↓	Li et al. (2025b)
Writer, Reader	WTAP, IGF2BP2	Oncogene	lnc-OXAR	Chemoresistance	WTAP/IGF2BP2→lnc-OXAR stability↑→Ku70 degeneration↓→facilitating DNA double-strand break (DSB) repair→oxaliplatin resistance	Lin et al. (2024)
Writer	METTL3	Oncogene	TRIM21 mRNA	Chemoresistance	METTL3→TRIM21 mRNA↓→G6PD↑→pentose phosphate pathway↑→oxaliplatin resistance	Jin et al. (2025)
Writer, Reader	METTL3, IGF2BP3	Oncogene	LARP4B mRNA	Targeted therapy resistance	METTL3/IGF2BP3→LARP4B↑→SPINK1↑→activating EGFR pathways→sorafenib resistance	Wang et al. (2024c)
Writer, Reader	METTL3, METTL14	Oncogene	SREBF2-AS1 transcript	Targeted therapy resistance	METTL3, METTL14→SREBF2-AS1 stability↑→SREBF2-AS1↑→recuiting FXR1 and TET1→SREBF2↑→sorafenib resistance	Wu et al. (2024)
Writer, Reader	METTL3, IGF2BP1	Oncogene	KIF9-AS1	Targeted therapy resistance	METTL3/IGF2BP2→stabilizing KIF9-AS1↑→SHOX2↑→sorafenib resistance	Yu et al. (2024b)
Writer, Reader	METTL3, YTHDF1	Tumor suppressor	FOXO3 mRNA	Targeted therapy resistance	METTL3/YTHDF1↓→FOXO3↓→sorafenib resistance	Lin et al. (2020)
Writer	RBM15B	Oncogene	TRAM2 mRNA	Targeted therapy resistance	RBM15B↑→TRAM2 mRNA stability↑→sorafenib resistance	Tan et al. (2022)
Reader	YTHDF2	Oncogene	—	Targeted therapy resistance	HSP90β→inhibiting ubiquitination of YTHDF2→YTHDF2↑→sorafenib resistance	Liao et al. (2023)
Writer	METTL3	Oncogene	USP15 mRNA	Targeted therapy resistance	METTL3→USP15 mRNA↑→LGALS3 stability↑→activating AKT/mTOR pathway↑→lenvatinib resistance	Fang et al. (2025)
Reader	IGF2BP3	Oncogene	PCK2 and NRF2 mRNAs	Targeted therapy resistance	Lactylated IGF2BP3→PCK2↑, NRF2↑→lenvatinib resistance in HCC.	Lu et al. (2024)
Reader	YTHDF1	Oncogene	NOTCH1 mRNA	Targeted therapy resistance	YTHDF1→NOTCH1 mRNA stability and translation→NOTCH1↑→lenvatinib and sorafenib resistance	Zhang et al. (2024)
Writer	METTL14	Oncogene	CHOP	Targeted therapy resistance	METTL14↓→CHOP↑→Regorafenib sensitivity↑	Pan et al. (2024)

### 4.1 Glycolysis

**FIGURE 2:**
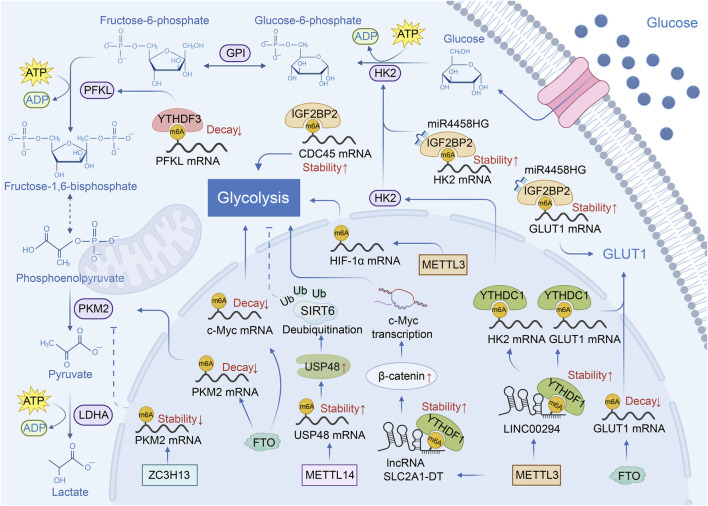
m6A RNA modification regulates glycolysis in HCC. The RNA m6A modification regulates glycolysis-related enzymes (GLUT1, HK2, PKM2) and other glycolysis-related proteins (USP48, β-catenin, SIRT6, c-Myc, HIF-1α, PFKL, CDC45) to influence the glycolysis of HCC. METTL14 and ZC3H13 inhibit glycolysis of HCC. METTL3, FTO, YTHDF3, and IGF2BP2 promote glycolysis of HCC.

Altered energy metabolism is an important hallmark of cancer. Specifically, the switch to glycolysis despite oxygen availability, referred to as aerobic glycolysis, is commonly observed in various cancers. Aerobic glycolysis, a phenomenon termed the Warburg effect, was first discovered in HCC and has been shown to be implicated in the pathogenesis of this malignancy (Feng et al., 2020). Recent studies indicate that m6A modification of glycolysis-related RNA regulates glycolysis in HCC (Figure 2). The m6A modification mediated by METTL14 maintains USP48 mRNA stability, consequently promoting the translation of USP48. Consequently, USP48 upregulates the stability of SIRT6 by K48-linked deubiquitination of SIRT6, which inhibits aerobic glycolysis (Du et al., 2021). Another research reported that SIRT6 inhibits the expression of *GLUT1* gene by encouraging deacetylation the H3K9 on its promoter to inhibit aerobic glycolysis and oncogenesis of HCC (Guo et al., 2022). On the contrary, upregulated by the HBXIP in HCC cells, METTL3 catalyzes the m6A modification of HIF-1α mRNA to promote the aerobic glycolysis (Fan et al., 2021). In addition, METTL3 mediates the m6A modification and stability of a glycolysis-related lncRNA called lncRNA SLC2A1-DT, which upregulates β-catenin expression. The elevated β-catenin activates the transcription of the glycolytic regulatory factor c-Myc, which encourages the glycolysis of HCC cells. Interestingly, the expression of METTL3 is driven by transcription factor c-Myc, creating a self-reinforcing cycle to promote glycolysis and accelerate HCC development (Zeng et al., 2024). In addition, ZC3H13 downregulates the stability of PKM2 mRNA through m6A modification, which reduces PKM2 protein levels. The reduced PKM2 level inhibits aerobic glycolysis and proliferation of HCC cells (Wang et al., 2021).

On the other hand, the m6A eraser FTO, facilitates aerobic glycolysis in HCC by reducing the mRNA degradation of glycolysis-related genes, including the *GLUT1*, *PKM2*, and *c-Myc* (Wang F. et al., 2023). Apart from m6A writers and erasers, m6A readers are also instrumental to the glycolysis of HCC cells. YTHDF3 inhibits the degradation of PFKL mRNA, thereby enhancing PFKL expression. As a key glycolytic enzyme, the elevated PFKL drives aerobic glycolysis and consequently enhances the proliferation, migration and invasion of HCC cells. (Zhou et al., 2022). In addition, CDC45 promotes the glucose consumption and lactate production by encouraging expression of PFKL (Wenjin et al., 2023). IGF2BP2 stabilizes the CDC45 mRNA through m6A modification to facilitate aerobic glycolysis in HCC (Wu et al., 2023). Moreover, interacting with miR4458 host gene, IGF2BP2 stabilizes the glycolytic enzyme, HK2 and GLUT1 mRNAs through m6A modification, thereby promoting glycolysis in HCC (Ye et al., 2023). It has also been observed that LINC00294 can promote aerobic glycolysis by facilitating the interaction of YTHDC1 with HK2, and GLUT1 mRNAs. Notably, the m6A modification mediated by METTL3/YTHDC1 enhances the stability of LINC00294 (Zhang et al., 2023).

In summary, all the above studies have shown that m6A modification is the main regulatory factor for aerobic glycolysis in HCC. Its main function is to regulate the post-transcriptional fate of a group of core glycolytic components. This regulation ultimately focuses on key glycolytic enzymes HK2, PKL1, and PKM2, the glucose transporter GLUT1 and upstream transcription factors. Particularly notable is the emergence of self-reinforcing regulatory loops, such as the METTL3/c-Myc positive feedback loop, which indicates that m6A can enable tumor cells to maintain a continuous glycolytic state. However, the main focus of this field is on the stability of mRNA. Whether m6A can dynamically and precisely regulate the translation efficiency of metabolic mRNA in response to rapid nutritional or energy signals has not been fully studied yet. Moreover, most of the evidence comes from cell line models and lacks validation in the complex *in vivo* tumor microenvironment.

### 4.2 Lipid metabolism

Aside from glycolysis, lipid metabolism is another important resource for cellular energy and the dysregulation of lipid metabolism is a prevalent event in cancer pathogenesis (Bian et al., 2021a). Given the liver’s central role in lipid metabolism, dysregulation of this process is critically involved in HCC tumorigenesis (Alannan et al., 2020). Recently, accumulating evidence has linked m6A RNA modification to the dysregulation of lipid metabolism in HCC (Peng et al., 2022). Excessive m6A modifications enhance the expression of ATP citrate lyase and stearoyl-CoA desaturase1, which results in increased cholesterol and triglyceride production, and lipid droplet accumulation in HCC (Yang et al., 2022). METTL3 promotes non-alcoholic fatty liver disease-mediated HCC by activating cholesterol biosynthesis. Briefly, METTL3 mediates the m6A modification of SCAP and then elevates the expression of SCAP. Consequently, increased SCAP promotes the secretion levels of cholesterol and cholesteryl esters, which impairs the function of CD8^+^ T cells, resulting in immune escape and HCC progression (Pan et al., 2023). Additionally, METTL3 stabilizes a lipogenesis-related lncRNA called LINC00958 through m6A modification and elevates the level of LINC00958 in HCC cells. LINC00958 drives lipogenesis by sponging miR-3619-5p to elevate the expression of hepatoma-derived growth factor, which promotes lipogenesis and malignant phenotypes of HCC (Zuo et al., 2020). Others promote lipid metabolism reprogramming, which is an early indicator of HCC malignancy (Cheng et al., 2018). ALKBH5-mediated m6A modification stabilizes the LINC01468 RNA and upregulates its level. LINC01468 activates the PI3K/AKT/mTOR signaling pathway by binding to the SH2 domain-containing inositol 5-phosphatase 2 and promoting cullin 4 A-linked ubiquitin decay, which increases *de novo* lipid biosynthesis and HCC progression (Wang H. et al., 2022).

**FIGURE 3:**
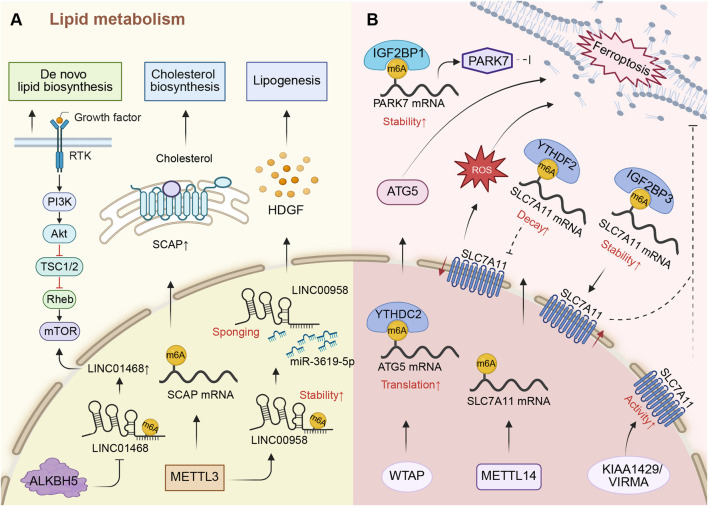
m6A RNA modification regulates lipid metabolism and ferroptosis in HCC. **(A)** METTL3 promotes cholesterol biosynthesis and lipogenesis by regulating the m6A modification of SCAP mRNA and LINC00958. ALKBH5 promotes de novo lipid biosynthesis by regulating m6A modification of LINC01468. **(B)** METTL14 and WTAP promote ferroptosis of HCC. KIAA1429/VIRMA, IGF2BP1, and IGF2BP3 inhibit ferroptosis of HCC. METTL14, KIAA1429/VIRMA, and IGF2BP3 regulate ferroptosis by influencing the level and activity of SLC7A11.

The evidence reviewed herein positions m6A modification as a pivotal epigenetic driver of lipogenic reprogramming in HCC, directly linking epitranscriptomic control to the hallmarks of metabolic disease and cancer. A clear mechanistic theme emerges that m6A writers and erasers exert influence by stabilizing key lipogenic effector mRNAs and oncogenic lncRNAs, which in turn activate master transcriptional regulators or signaling cascades like PI3K/AKT/mTOR to fuel *de novo* lipid synthesis (Figure 3A). Notably, the finding that METTL3-mediated cholesterol synthesis impairs CD8^+^ T cell function reveals a crucial crosstalk between metabolic reprogramming and immune evasion, suggesting m6A acts at a critical nexus coordinating tumor metabolism and microenvironment.

### 4.3 Ferroptosis

Ferroptosis was introduced in 2012 to denote a form of iron-dependent, non-apoptotic cell death (Jiang et al., 2021; Tang et al., 2021). This distinct cell death pathway is mediated through excessive lipid peroxidation and has been implicated in the pathogenesis of diverse diseases, including cancer (Mou et al., 2019; Yang et al., 2021). Recently, some studies have indicated that ferroptosis is modulated by m6A regulators and is involved in HCC progression (Figure 3B). As an m6A writer, WTAP, with the help of YTHDC2, increases the m6A modification on ATG5 mRNAs, resulting in augmented ATG5 protein levels. This facilitates ATG5-dependent ferroptosis and HCC development (Li Y. et al., 2024). In addition, the cystine/glutamate antiporter termed system xc-, consists of cystine/glutamate antiporter SLC7A11 and SLC3A2, is a crucial part of antioxidant system in cells (Cao and Dixon, 2016). It facilitates GSH synthesis, thereby reducing ROS levels. Conversely, inhibiting SLC7A11 promotes ROS accumulation and ferroptosis (Li et al., 2020). METTL14-catalyzed m6A modification of SLC7A11 mRNA promotes its degradation via YTHDF2 recognition, thereby resulting in decreased SLC7A11 protein expression. The decreased SLC7A11 stimulates ROS production and eventually ferroptosis. Thus, YTHDF2 and METTL14 may play an anti-tumor role in HCC. Additionally, a study reported that interventional embolization of HCC causes hypoxia and upregulates the level of HIF-1α, which inhibits METTL14 to prevent ferroptosis and support HCC progression (Fan et al., 2021). On the contrary, KIAA1429 increases the activity of the SLC7A11 through m6A modification to protect HCC cells from ferroptosis. It has been shown that inhibition of KIAA1429 halts the growth of HCC (Wang H. et al., 2023). IGF2BP3 is recruited by LINC00942 to increase the stability of SLC7A11 mRNA, which upregulates the level of SLC7A11 (Jin et al., 2024). This suppresses ferroptosis in HCC and promotes HCC progression. IGF2BP1, on the other hand, is recruited by circCMTM3 to reinforce the stability of PARK7 mRNA. PARK7 has the function of suppressing ferroptosis in that it stabilizes the functional integrity of S-adenosyl homocysteine hydrolase (Cao et al., 2020). Hence, the upregulated PARK7 by m6A modification inhibits ferroptosis and ultimately reinforces HCC carcinogenesis (Zhao et al., 2023).

The emerging interplay between m6A modification and ferroptosis outlines a sophisticated regulatory axis in HCC (Figure 3B), where epitranscriptomic factors decisively influence cellular redox fate. A central conflict is evident. Regulators like METTL14/YTHDF2 and WTAP/YTHDC2 can promote ferroptosis by suppressing SLC7A11 or enhancing ATG5, acting as tumor suppressors. Conversely, KIAA1429 and IGF2BP1/3 exert anti-ferroptotic effects to support tumor survival. This context-dependent duality suggests that the overall impact of m6A on ferroptosis is not predetermined but is dynamically shaped by the specific molecular and microenvironmental context, such as hypoxia modulating METTL14.

### 4.4 Cancer stemness

**FIGURE 4:**
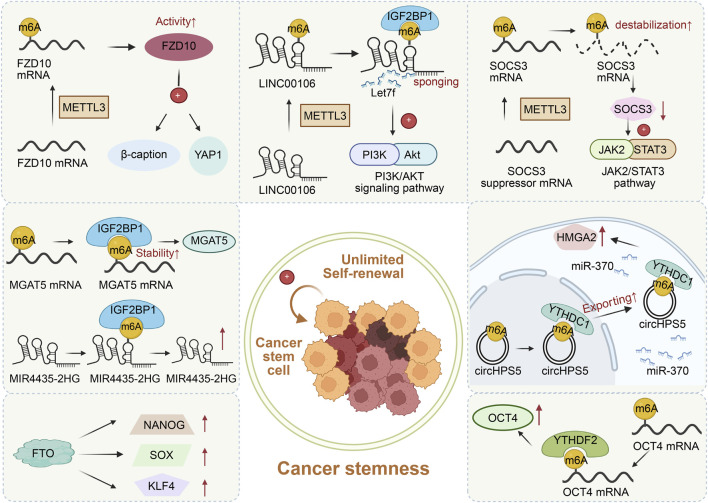
m6A RNA modification regulates cancer stemness of HCC. METTL3 promotes the cancer stemness of HCC by regulating the m6A modification of FZD10 mRNA, LINC00106, and SOCS3 mRNA. FTO upregulates cancer stemness-related proteins. YTHDF2, YTHDC1, and IGF2BP1 respectively identify the m6A modification of OCT4 mRNA, circHPS5, MGAT5 mRNA and lncRNA MIR4435-2HG and promote the cancer stemness of HCC.

Cancer stemness is defined by the population of cancer cells with stem-like (CSCs) properties such as self-renewal and tumor-initiation potential (Katoh and Katoh, 2022). CSCs are notorious for facilitating tumor initiation, metastasis, and tumor relapse (Chen and Cheng, 2022; Chen et al., 2021; Walcher et al., 2020). In the past 2 decades, there have been increasing efforts to characterize liver cancer stem cells (LCSCs) (Lee et al., 2022). A well-acknowledged observation is that LCSCs promote different aspects of HCC pathogenesis, from initiation and growth to metastasis and treatment resistance (Lv et al., 2021). Yet, the underlying mechanisms of LCSCs are not well understood. Emerging evidence implicates m6A modifications in regulating cancer stemness properties in HCC (Figure 4). METTL3-mediated m6A methylation of FZD10 mRNA leads to the activation of YAP1 and β-catenin to facilitate self-renewal, tumorigenesis, and metastasis of LCSCs (Wang J. et al., 2023). Additionally, METTL3 promotes the stemness of HCC cells by upregulating LINC00106, a newly found lncRNA, in an m6A-dependent manner (Liang et al., 2021). METTL3 also destabilizes SOCS3 mRNA, which downregulates SOCS3 protein levels. This alleviates the suppression of SOCS3 on JAK2/STAT3 pathway and eventually results in the JAK2/STAT3 pathway-dependent increase in LCSC stemness (Hu et al., 2024). In addition, AMD1-mediated upregulation of SPD promotes the interaction between IQGAP1 and FTO, which increases the phosphorylation but decreases the ubiquitination of FTO, resulting in elevated levels of FTO. The increase in FTO levels upregulates the expression of key pluripotency factors that induce self-renewal such as NANOG, SOX2, and KLF4 to promote the stemness of HCC cells (Bian et al., 2021b). Besides, YTHDF2 sustains the LCSC phenotype by enhancing the m6A methylation of another pluripotency factor OCT4 mRNA (Wang and Kang, 2018; Zhang et al., 2020). YTHDC1 increases the cytoplasmic output of m6A-modified circHPS5, which acts as a sponge for miR-370 to control the expression of an important protein that supports stemness of tumor cells called HMGA2, thereby promoting CSC phenotypes (Mansoori et al., 2021; Rong et al., 2021). IGF2BP1 increases the stability of MGAT5 mRNA through m6A, which also enhances the LCSC phenotype in HCC (Yang et al., 2021). The stemness in HCC is also a consequence of lncRNA MIR4435-2HG upregulation, which is due to the IGF2BP1-mediated m6A modifications of MIR4435-2HG (Zhu et al., 2023).

In short, the m6A modification maintains the stemness characteristics of LCSCs by constructing a multi-level epigenetic regulatory network (Figure 4). This regulatory network mainly acts on three key levels. Firstly, the m6A modification can directly stabilize the mRNAs of core pluripotency factors such as OCT4 and NANOG, thereby maintaining the self-renewal ability of stem cells. Secondly, this modification affects cell fate decisions by regulating key signaling pathways such as YAP/β-catenin and JAK2/STAT3. Moreover, m6A is also involved in reshaping the regulatory network of non-coding RNAs such as LINC00106 and circHPS5. Particularly important is that the metabolic-mediated regulatory mechanism adds a new dimension to this network. For example, the SPD-AMD1 axis links intracellular polyamine metabolism with the epigenetic transcriptional regulation of stem cell characteristics by stabilizing the FTO demethylase, revealing the functional coupling between metabolic reprogramming and epigenetic regulation.

### 4.5 Tumor immunity

The immune system dysregulation is critically implicated in the development of cancers (Barbosa et al., 2021). While some studies indicate that the immune system can inhibit cancer cell growth, others have shown that a dysfunctional immune system may actually promote tumor progression (Obeagu, 2025). A growing number of studies show that m6A modifications impact the development and progression of HCC by influencing tumor immunity (Chen X. et al., 2025). T cells are the primary anti-tumor immune cells and are part of the acquired immunity (Cachot et al., 2021). Programmed death-1 (PD-1) is a cell surface receptor that inhibits T cell activation by binding with its ligand called programmed death-ligand 1 (PD-L1) (Jiang et al., 2019). Under physiological conditions, PD-1 resides on activated T cells, while its ligand PD-L1 is displayed by antigen-presenting cells such as dendritic cells and macrophages. Their interaction is mainly to ensure that the immune system is activated only at the right time and to minimize chronic autoimmune inflammation (Keir et al., 2008). However, PD-1 signaling is commonly hijacked by cancer cells to escape immune surveillance (Yi et al., 2022). In HCC, WTAP enhances the stability of circCCAR1 with m6A modifications, which allows circCCAR1 to be secreted by HCC cells with the help of hnRNPA2B1. The secreted circCCAR1 is taken up by CD8^+^ T cells, leading to the stabilization of PD-1 and the eventual dysfunction of CD8^+^ T cells. Moreover, WTAP, which is upregulated by circCCAR1, sponges miR-127-5p to form a positive feedback loop that promotes the growth and metastasis of HCC (Hu et al., 2023). YTHDF2 can enhance the translation of ETV5 mRNA by recognizing the m6A modifications in its 5′-untranslated region. The upregulated ETV5 induces PD-L1 transcription to encourage HCC immune escape (Wen et al., 2024).

In addition to acquired immunity, innate immunity can also be regulated by m6A modification to influence HCC tumorigenesis (Kim et al., 2021). Macrophages that exhibit remarkable diversity and plasticity are crucial for innate immunity (Wang Y. et al., 2019; Zhao et al., 2023). They can be polarized into M1 and M2 phenotypes in extreme states. M1 macrophages are induced by microbial products or pro-inflammatory cytokines such as interferon-γ to promote inflammation (Chen et al., 2023). In tumor tissues, the M2 macrophages, induced by cytokines (including IL-4, IL-13, IL-10) modulate adaptive immunity to augment tumor development (Mantovani et al., 2002). In HCC, METTL3 activates RNA-binding protein 14 mRNA via m6A modification with the help of YTHDF1, which stimulates the polarization of Kupffer cells into the M2 phenotype, thereby encouraging HCC tumorigenesis (Hu et al., 2022). In addition to that, METTL3 and METTL16 enhance the stability of lncRNA ZNNT1, leading to the upregulation of ZNNT1 levels in an m6A-dependent manner. The elevated ZNNT1 promotes the expression and secretion of osteopontin, which facilitates the recruitment and polarization of macrophages into M2 macrophages. In turn, M2 macrophages secrete S100A9 to upregulate ZNNT1 expression in HCC cells through AGER/NF-κB signal transduction to promote the malignant progression of HCC by encouraging HCC cell growth, invasion and migration (Wei et al., 2024).

Moreover, myeloid-derived suppressor cells (MDSCs) constitute an immunosuppressive population of pathologically activated immature neutrophils and monocytes, which suppress T cell function and drive immune escape in malignancies (Veglia et al., 2018; Veglia et al., 2021; Wu Y. et al., 2022). In HCC, YTHDF1 combines with enhancer of EZH2 mRNA modified by m6A and promotes the translation of EZH2. Highly expressed EZH2 promotes the expression and secretion of IL-6, and the recruitment and activation of MDSCs, leading to the dysfunction of CD8^+^ T cells and the occurrence of NASH-dependent HCC (Wang et al., 2023e). Collectively, m6A modification profoundly influences anti-tumor immunity in HCC by regulating T cell functionality and MDSC recruitment. Notably, this regulatory network extends beyond immune cells to structural components within the tumor microenvironment, such as cancer-associated fibroblasts (CAFs). Recent work has demonstrated that CCL15 derived from HCC cells activates STAT3 signaling in CAFs, markedly upregulating expression of the m6A demethylase FTO. FTO subsequently promotes secretion of the pro-tumorigenic factor CXCL5 by erasing m6A modifications on CEBPA mRNA, thereby driving HCC cell proliferation and establishing a positive feedback loop. These findings illustrate that m6A modification serves as a central communication hub linking tumor cells with stromal cells such as CAFs, facilitating HCC progression through remodeling of the molecular ecosystem within the microenvironment (Wang et al., 2024b).

**FIGURE 5:**
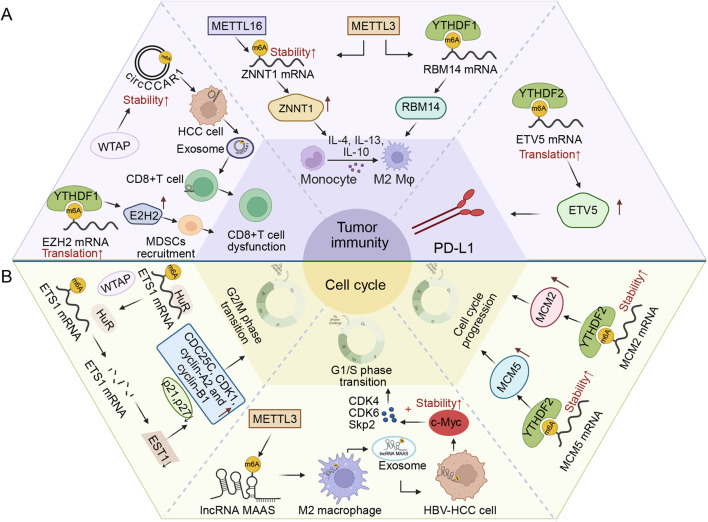
m6A RNA modification regulates tumor immunity of HCC and cell cycle progression of HCC cells. **(A)** METTL3, METTL16, WTAP, YTHDF1, and YTHDF2 suppress tumor immunity of HCC through different mechanisms. METTL3 and METTL16 promote the differentiation of monocytes to M2 macrophages and tumor immunosuppression. WTAP and YTHDF1 induce the dysfunction of CD8+T cells and impair anti-tumor immunity. YTHDF2 upregulates PD-L1 and induces tumor immune escape. **(B)** METTL3, WTAP, and YTHDF2 promote the progression of the cell cycle in HCC cells in an m6A modification-dependent way. METTL3 promotes the G1/S phase transition of HCC in an m6A-dependent way. WTAP promotes G2/M phase transition by regulating cell cycle-related proteins. YTHDF2 promotes the progress of the cell cycle by increasing the stability of MCM2 and MCM5 mRNAs.

In summary, the m6A modification emerges as an essential regulator of the immunosuppressive tumor microenvironment in HCC, orchestrating dysfunction across both adaptive and innate immunity (Figure 5A). It facilitates immune escape through diverse and complementary mechanisms, including impairing CD8^+^ T cell function via the PD-1/PD-L1 axis and exosomal circRNAs, polarizing macrophages towards a pro-tumor M2 phenotype, and expanding MDSC populations. The described positive feedback loops such as WTAP/circCCAR1 and ZNNT1/S100A9 suggest that m6A can establish and stabilize an immunosuppressive niche, making it a potential linchpin in tumor-immune crosstalk.

### 4.6 Cell cycle progression

Abnormal cell cycle progression, controlled by checkpoints and associated with the sequential activation of cyclin-dependent kinases, is a fundamental mechanism that regulates tumorigenesis (Icard et al., 2019; Liu et al., 2022). It is found that m6A modification can regulate the cell cycle progression of HCC to influence the initiation and development of HCC. WTAP impairs ETS1-mediated cell cycle regulation by disrupting the interaction between Hu-Antigen R and tumor suppressor ETS1 mRNA in an m6A-dependent way, thereby promoting HCC tumorigenesis. Notably, WTAP knockdown upregulates p21 and p27 while downregulating CDC25C, CDK1, cyclin-A2, and cyclin-B1, ultimately inducing G2/M phase arrest (Chen et al., 2019). Additionally, HBV-related HCC secretes HBeAg to enhance METTL3-mediated m6A modification on the lncRNA MAAS, thereby increasing the level of MAAS in M2 macrophages. The elevated MAAS is subsequently assembled into exosomes to enter HBV-HCC cells and stabilizes the c-Myc protein, which facilitates the G1/S transition by activating the transcription of CDK4, CDK6, and S-phase kinase-associated protein 2. These ultimately result in the aberrant proliferation of HBV-HCC (Tao et al., 2022). In eukaryotes, MCMs initiate DNA replication by participating in an important G1 process called origin licensing (Li et al., 2023; Petropoulos et al., 2019). This process prepares the cell for the S phase by loading enough MCMs at DNA replication origins (Matson et al., 2019). They serve as reliable cell cycle-based prognostic and early tumor biomarkers (Tachibana et al., 2005). Yang et al. discovered that YTHDF2 could enhance the stability of MCM2 and MCM5 transcripts via m6A modifications, which encourages the cell cycle and the tumorigenesis of HCC related to HBV infection. The stability and oncogenic activity of YTHDF2 are enhanced following HBV infection by increasing O-GlcNAc transferase-mediated O-GlcNAcylation of YTHDF2 (Yang et al., 2023).

Collectively, m6A modification precisely regulates the cell cycle process through targeted strategies in HCC (Figure 5B). Its core objective is to circumvent key checkpoint restrictions and drive malignant proliferation. This regulatory mechanism is mainly manifested as the degradation of tumor suppressive mRNAs and the stabilizing effect on replication factors and positive regulators of the cell cycle. The downregulation of ETS1 and the maintenance of minichromosome maintenance complex and cell cycle proteins are typical examples. It is worth noting that the hepatitis B virus introduces the interaction between the virus and the host into this regulatory network by enhancing the functional activity of the METTL3 writer enzyme and the YTHDF2 reader enzyme, suggesting that the pathogenic mechanism of the virus may partially rely on the hijacking of the host’s epigenetic transcriptome to achieve continuous cell cycle progression. From a therapeutic perspective, the high expression state of replication factors caused by m6A-dependent overexpression brings specific vulnerabilities to the cells, which provides potential intervention targets for developing synergistic treatment strategies for hepatitis B-related hepatocellular carcinoma.

### 4.7 Therapy resistance

**FIGURE 6:**
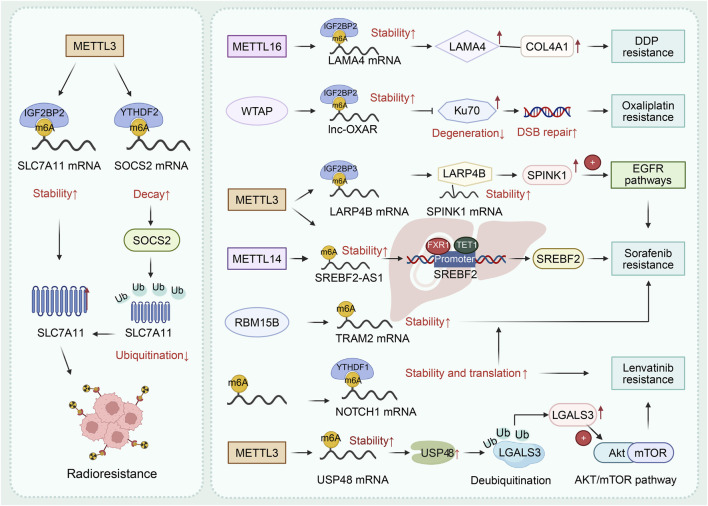
m6A RNA modification regulates therapy resistance of HCC. METTL3 induces the radioresistance by regulating SLC7A11 mRNA and SOCS2 mRNA with the help of IGF2BP2 and YTHDF2. METTL16 promotes DDP resistance by enhancing the stability of LAMA4 mRNA aided by IGF2BP2. WTAP stabilizes lnc-OXAR to induce oxaliplatin resistance. METTL3, METTL14, RBM15B and YTHDF1 regulate the sorafenib resistance by m6A mediated RNA modification. YTHDF1 and METTL3 promote lenvatinib resistance by regulating m6A modification of NOTCH1 mRNA and USP48 mRNA respectively.

Resistance to therapy remains a major obstacle in oncology (Zhuang et al., 2023). Recent research has established m6A RNA modification as a key regulator of therapy resistance in HCC (Figure 6). The m6A regulators influence resistance to radiotherapy, chemotherapy and targeted therapy by fine-tuning the expression of critical genes (Fu et al., 2024; Wang J. et al., 2023; Xu et al., 2020). In terms of radioresistance, METTL3 promotes radioresistance in HCC cells by up-regulating SLC7A11 expression and inhibiting SOCS2-mediated SLC7A11 protein ubiquitination (Zhang C. et al., 2025). In chemotherapy resistance, METTL16 and IGF2BP2 collectively promote cisplatin resistance by regulating LAMA4 mRNA stability in an m6A-depednt way and upregulating COL4A1 (Cao and Bi, 2025). In contrast, WTAP deficiency impairs FOXM1 stability, preventing DNA damage responses to sensitized HCC cells to cisplatin both *in vitro* and *in vivo* (Huang et al., 2025). In addition, METTL3 silencing repressed lncRNA TUG1 in an m6A-dependent manner, a process mediated by the miR-9/EIF5A2 axis to confer doxorubicin sensitivity (Li E. et al., 2025). For oxaliplatin resistance, WTAP/IGF2BP2 stabilizes lnc-OXAR through an m6A-dependent mechanism, which drives oxaliplatin resistance in NASH-HCC by recruiting Ku70 and CSTA to inhibit Ku70 degradation and repair DNA double-strand breaks (Lin et al., 2024). Similarly, METTL3 destabilizes TRIM21 mRNA in an m6A-YTHDF2-dependent manner, weakens TRIM21-driven ubiquitination degradation of G6PD, thereby activating the PPP pathway and promoting oxaliplatin resistance in HCC (Jin et al., 2025).

As for targeted therapy resistance, sorafenib resistance involves multiple m6A regulators. With the help of IGF2BP3, METTL3 upregulates expression of LARP4B, which stabilizes SPINK1 mRNA to activate EGFR signaling, thereby supporting sorafenib resistance in HCC (Wang et al., 2024c). Additionally, METTL3/METTL14-mediated m6A modification stabilizes the SREBF2-AS1 transcript, which in turn recruits FXR1 and TET1 to demethylate the SREBF2 promoter, ultimately upregulating SREBF2 to drive tumor progression and sorafenib resistance (Wu et al., 2024). METTL3/IGF2BP1-driven stabilization of lncRNA KIF9-AS1 drives sorafenib resistance by facilitating USP1-mediated deubiquitination and stabilization of SHOX2 (Yu Y. et al., 2024). Conversely, depletion of METTL3 under hypoxia promotes sorafenib resistance in HCC by abolishing YTHDF1-dependent stabilization of FOXO3 mRNA (Lin et al., 2020). Moreover, RBM15B induces sorafenib resistance through m6A-dependent stabilization of TRAM2 mRNA (Tan et al., 2022). In addition, The HSP90β/YTHDF2 axis is critically involved in sorafenib resistance, wherein HSP90β stabilizes the YTHDF2 protein by inhibiting its STUB1-mediated degradation (Liao et al., 2023). For lenvatinib resistance, METTL3-mediated m6A modification upregulates USP15, which in turn deubiquitinates and stabilizes LGALS3 to activate the AKT/mTOR pathway, ultimately driving lenvatinib resistance in HCC (Fang et al., 2025). In lenvatinib-resistant HCC, a novel positive feedback loop is established. Lactate-driven lactylation of the m6A reader IGF2BP3 enhances the stability of PCK2 and NRF2 mRNAs, which in turn reprograms serine metabolism to supply SAM, further amplifying their own m6A modification and fortifying antioxidant defense to sustain resistance (Lu et al., 2024). By enhancing NOTCH1 expression in an m6A-dependent manner, YTHDF1 promotes resistance to lenvatinib and sorafenib (Chen A. et al., 2024). Similarly, METTL14 enhances regorafenib resistance by up-regulating CHOP (Pan et al., 2024).

In general, m6A modification plays a critical role in the adaptive treatment resistance of HCC, and it functions through a series of distinct and specific mechanisms (Figure 6). A convergent theme emerging from these disparate resistance mechanisms is the m6A-mediated stabilization of key pro-survival mRNAs, frequently culminating in self-reinforcing feedback loops that lock the tumor cell into a drug-resistant state. As illustrated across therapies (Figure 6), m6A regulators commonly act to enhance the mRNA stability and expression of effector proteins. These effectors then activate compensatory pathways to sustain proliferation under therapeutic pressure. Crucially, several of these pathways feed back to upregulate the m6A machinery itself, creating a resilient and perpetuating resistance network. Therefore, beyond diverse downstream effects, the core unifying pathway is the m6A-dependent post-transcriptional enforcement of adaptive survival programs, making the m6A epitranscriptome a central integrator of resistance across multiple therapies.

## 5 Therapy approaches targeting m6A RNA modification in HCC

Given the pivotal role of m6A modification in HCC pathogenesis and progression, targeting this epitranscriptomic machinery has emerged as a promising therapeutic strategy. Current approaches primarily focus on small-molecule inhibitors and nano-delivery systems.

### 5.1 Small molecule inhibitors

Dysregulated m6A regulators are recognized as oncogenic drivers in HCC, spurring the development of small-molecule inhibitors designed to block their catalytic activity or disrupt functional integrity (Ma et al., 2024). So far, inhibitors of METTL3, FTO and multitargeted METTL have been reported. METTL3 small molecule inhibitors were first screened by Bedi et al. from a library of 4000 adenosine analogues and derivatives using a cofactor simulation approach, with one compound having the most favorable inhibitory potency (Bedi et al., 2020; Zeng et al., 2020) (IC50 = 8.7 μM). STM2457 targets METTL3 with high potency and selectivity, suppressing the progression of AML (Yankova et al., 2021), ICC (Xiong et al., 2022), prostate cancer (Haigh et al., 2022), and SHH-medulloblastoma (Zhang et al., 2022) by lowering mRNA m6A levels. Especially, STM2457 enhance the sensitivity of tumors to lenvatinib in multiple mouse HCC models (Wang et al., 2023d). In addition, the natural product quercetin suppresses HCC cell proliferation by acting as a METTL3 inhibitor. It functions by occupying the pocket of SAM’s adenosine moiety, thereby inhibiting the enzyme’s activity (Du et al., 2022). It is worth mentioning that an oral small molecule METTL3 inhibitor named STC-15 has been undergoing clinical trials. Researchers completed Phase I clinical trials evaluating safety, pharmacokinetics, and antitumor activity in advanced solid tumors, but the specific results have not yet been announced (Eleftheriou et al., 2025). Furthermore, Rhein, identified as the first FTO inhibitor via structure-based virtual screening, competitively binds to the enzyme’s catalytic domain, thereby suppressing its demethylase activity (Chen et al., 2012). The newly discovered FTO inhibitor CS1 potentiates anti-PD-1 therapy, exhibiting superior tumor suppression in HCC models through a synergistic mechanism (Chen A. et al., 2024). Then, the latest study identified two first-in-class, highly potent multi-target inhibitors (ZINC70666503 and ZINC13000658) through computer-aided design. They concurrently target the catalytic domains of key METTL methyltransferases, including METTL1, METTL3, METTL6, METTL16, and METTL18. Among them, ZINC13000658 demonstrated significant anti-proliferative efficacy in HCC cell lines, supporting the promise of this multi-targeting strategy (Morshed et al., 2025).

### 5.2 Nano-delivery system

Nanotechnology offers a robust platform for precision drug delivery in HCC. Lipid nanoparticles (LNPs) (Ebrahimi et al., 2023) and polymer nanoparticles (NPs) (Wang B. et al., 2024) enable efficient encapsulation and delivery of therapeutic agents, such as siRNAs targeting m6A regulators. It was the application of LNP-encapsulated small-interfering Ythdf1 that significantly improved the efficacy of anti-PD-1 therapy in NASH-HCC allograft models (Wang et al., 2023e). Apart from that, there is a new type of nanomedicine MFMP, which is used in combination with radiofrequency ablation (RFA) to treat HCC. It is composed of several components such as MnO_2_ (manganese dioxide), FIDAS-5 (a MAT2A inhibitor), macrophage cell membrane and anti-PD-L1. By simultaneously blocking the PD-L1 immune checkpoint, using the released FIDAS-5 for epigenetic regulation (activating cGAS and inhibiting EGFR), and enhancing the STING immune pathway with Mn^2+^, the immune system is activated through multiple approaches, thereby effectively inhibiting tumor recurrence and metastasis and establishing immune memory (Li X. et al., 2024). It is worth noting that there is a pH-responsive lipid nanoparticle (Lip@si-YTHDF2) that delivers a highly efficient siRNA targeting YTHDF2 (Guo et al., 2025). This nanoparticle can respond to the acidic environment characteristic of the tumor microenvironment, facilitating the selective release at the tumor site while protecting normal tissues. This provides a possible solution to the toxicity problems caused by systemic drug administration.

## 6 Conclusion and perspectives

The high mortality and morbidity rates associated with HCC necessitate the development of improved diagnostic and therapeutic strategies. The m6A RNA modification has become a focus of cancer research, particularly in HCC. This modification is controlled by three types of regulators, and their combined efforts result in a dynamic system that governs cellular metabolism and functionality. Any dysfunction in this dynamic m6A system, including overexpression, lowered expression, and specific alterations of m6A regulators, could potentially contribute to the development of HCC due to their critical roles in aerobic glycolysis, lipid metabolism, ferroptosis, cancer stemness, tumor immunity, cell cycle progression and therapy resistance. While recent studies have primarily concentrated on the role of individual m6A regulators in HCC, it is important to remember that m6A modification is a system in which “writers”, “erasers”, and “readers” work in synergy. The interplay between multiple regulators in the mechanisms of HCC remains to be clarified. As research progresses, the role of m6A in predicting and treating HCC continues to be uncovered. Various methods targeting m6A regulators, including inhibitors, agonists, gene editing, and nanoparticles, have demonstrated significant potential in treating HCC. However, these findings are largely confined to preclinical studies in cells and animal models, with clinical evidence remaining scarce. Further clinical trials are necessary to establish their applicability in human patients.

Although the critical roles of m6A modification in HCC pathogenesis have been widely established, the complexity of its regulatory network and the pathways for clinical translation remain largely unexplored, presenting numerous unresolved questions. At the mechanistic level, existing works mostly focus on the functional interpretation of individual m6A regulatory factors. In the future, more emphasis should be placed on analyzing the integrity, contextual dependence and spatio-temporal dynamic characteristics of its regulatory network from a systemic perspective. Integrating m6A epigenomic transcriptomics with multi-dimensional data such as genomics and proteomics will help construct a more complete pathogenic network of HCC, thereby identifying the core pathways and key targets that drive tumor progression. In addition, the function of m6A has significant cell type specificity (Gao et al., 2022). It often shows immunosuppressive effects in tumor cells, while in immune cells such as T cells and myeloid cells, it directly participates in the shaping of anti-tumor immune responses (Wang H. et al., 2019; Xiong et al., 2022). Using cell-specific gene knockout models to accurately analyze the functional differences of m6A in each component of the microenvironment will be an important way to understand its multi-dimensional regulatory mechanism and avoid the side effects of systemic intervention.

From a technological standpoint, there is still a lack of technical systems that can realize real-time, *in situ* and non-invasive monitoring of m6A modification at the tissue or *in vivo* level. The development of highly sensitive molecular probes, combined with multimodal imaging techniques, will provide the possibility for the localization of m6A enrichment areas during surgery and the dynamic prediction of postoperative risks. Furthermore, the continuous advancements in single-cell sequencing and spatial transcriptomics technologies are providing new directions for HCC-related research. Single-cell sequencing can reveal the gene expression status of individual cells, reflect the heterogeneity among cells, and deeply understand the expression regulatory mechanisms during cell growth (Choi et al., 2020). Currently, studies have used single-cell sequencing methods to find significant enrichment of IGFBP3 in astrocytes in HCC tissues, and in-depth research has deepened the understanding of the HCC immune microenvironment and provided directions for HCC immunotherapy (Chen S. et al., 2025). Spatial transcriptomics has also been used to reveal the heterogeneity within HCC tumors and the spatial expression patterns in the immune microenvironment (Wang Y. F. et al., 2022). The integration of single-cell transcriptomics and spatial transcriptomics has revealed the fibroblast subtypes in hepatocellular carcinoma, including spatial distribution, differentiation trajectory, and therapeutic potential (Liu et al., 2025). Looking to the future, the integration of single-cell m6A epitranscriptome sequencing and spatial transcriptomics is expected to fundamentally reshape our understanding of HCC heterogeneity within the next 5 years. This integration could overcome the limitations of current bulk sequencing methods, which struggle to reveal epigenetic differences at the single-cell level. The single-cell technology will analyze the unique m6A “fingerprints” of malignant clones, stem cells, and drug-resistant transitional cells, and reveal the m6A-based interaction mechanisms between tumors and the microenvironment. Meanwhile, the spatial technology will provide the tissue context for these discoveries, clarifying the distribution patterns of m6A modifications at key sites such as the invasion front and immune microregions. The integration of these two technologies not only enables the discovery of spatially resolved m6A markers for more accurate prognosis prediction, but also locates the treatment-vulnerable targets dependent on specific modification enzymes. Ultimately, it will drive HCC research to shift from the traditional genetic two-dimensional model to a new four-dimensional paradigm integrating genetics, epigenetics, space, and temporal dynamics, providing a new foundation for precision diagnosis and treatment.

Clinical transformation is the ultimate manifestation of the research value of m6A. Although numerous studies have confirmed that m6A regulators have the potential to serve as non-invasive diagnostic markers such as liquid biopsy, their transition to routine clinical applications still faces multiple fundamental challenges. These challenges mainly involve technical methods, biological complexity, and clinical validation. There are still significant limitations at the technical level. Currently, detection methods based on antibodies or chromatography-mass spectrometry have insufficient sensitivity, are complex to operate, and have a low degree of standardization, making it difficult to achieve stable and repeatable detection in complex clinical samples such as trace and fragmented circulating tumor RNA. These methods are costly, have limited throughput, and have strict requirements for the expertise of the operators, which is not conducive to large-scale clinical screening (Yan et al., 2025). Moreover, most of the existing technologies are unable to meet the clinical requirements for specificity, dynamic monitoring, and spatial resolution. Because they cannot effectively distinguish highly homologous regulatory enzyme subtypes, observe real-time dynamic changes of m6A in living cells, or achieve precise positioning at the single-cell and subcellular levels (Chen et al., 2026; Shi et al., 2026). Besides, the biological characteristics of m6A modification itself also bring about interpretation difficulties. This modification is highly dynamic, cell type-dependent, and site-specific, and is easily influenced by various physiological and pathological factors. Moreover, there is significant heterogeneity within tumors (Shi et al., 2026). At the same time, there is functional redundancy and compensatory effect among key regulatory factors, and due to its relatively conservative evolution, it is difficult to accurately reflect disease specificity through the detection results targeting a single target (Chen et al., 2026; Shi et al., 2026). This complexity makes it challenging to establish stable and universal modification thresholds or patterns as reliable diagnostic criteria. Additionally, the clinical translation pathway is not yet fully developed. Currently, there is still a lack of large-scale, multi-center, prospective studies that have been rigorously designed to verify the diagnostic efficacy of specific m6A markers or combinations. Converting laboratory methods into standardized, high-throughput detection processes suitable for clinical use is also a major gap (Chen et al., 2026). Future translational research needs to focus on developing cost-effective, easy-to-operate, and rigorously validated detection platforms, and systematically evaluate their efficacy in real clinical scenarios, in order to truly promote the practical application of m6A biomarkers (Yan et al., 2025).

As the research progresses, the understanding of the functions of m6A has shifted from a single modification layer to an integrated perspective of “interacting epigenetic transcriptome”. The latest studies have shown that m6A does not act independently but forms a close regulatory dialogue with other key modifications. For instance, the histone modification H3K36me3 can specifically induce m6A modification in the oncogenic lncRNA L1CAM-AS1, stabilizing the RAN protein and activating the NF-κB signal, driving macrophage M2 polarization and leading to immune therapy resistance (Wang T. et al., 2025). This reveals the “histone code-m6A-tumor immune microenvironment” cascade regulatory axis. At the same time, systematic multi-omics analysis has found that in liver cancer, m6A interacts extensively with the regulators of m5C, and the two jointly affect disease progression, treatment response, and immune evasion, forming a higher-dimensional epigenetic - epigenetic transcriptional collaborative network (Tian et al., 2023). These findings emphasize that future research should focus on elucidating the dynamic interaction mechanisms between m6A and histone modifications, as well as other DNA or RNA modifications, especially in the specific pathways and targets in tumor immune regulation. This will provide a new perspective for developing combined epigenetic intervention strategies and reversing immune therapy resistance.

In terms of therapeutic strategies, small molecule regulators targeting “writers”, “erasers” and “readers” have become a research and development hotspot. For instance, ALKBH5 inhibitor ALK-04, YTHDF1 inhibitor Tegaserod, YTHDF2 inhibitor DC-Y13-27, IGF2BP1/2 inhibitor AVJ16 and CWI1-2, etc., have been reported successively, but their efficacy and mechanism in HCC still need to be verified (Wei et al., 2025). In addition, the systemic toxicity of these targeted drugs is rarely evaluated, so it is necessary to evaluate their safety for clinical application. It is worth noting that systemic administration of m6A modulators may cause off-target effects (Jiang et al., 2025). Current research on liver-specific delivery systems is also making continuous progress. The delivery systems functionalized with triantennary N-acetylgalactosamine (GalNAc), including mesoporous silica nanoparticles (Cordeiro et al., 2022) and metal-organic framework (Wan et al., 2025), have a high affinity for the overexpressed subunit glycoprotein receptor in HCC cells. This provides the possibility for achieving targeted liver-specific delivery and more precise treatment. However, this research has not yet been combined with m6A modification. Therefore, further development of liver-specific nanodelivery systems and combination with pH or enzyme-responsive controlled-release mechanisms can significantly enhance drug accumulation at the tumor site and achieve efficient and low-toxicity therapeutic effects. In addition, the precise editing platform based on CRISPR/dCas13 also provides a brand-new tool for site-specific m6A modification regulation (Yu L. et al., 2024).

Finally, the limitations associated with monotherapy in HCC treatment have driven the development of combination strategies as a promising future direction, with the central approach focusing on enhancing the efficacy of existing therapies through modulation of m6A. Given that m6A modification plays a critical role in mediating resistance to radiotherapy and chemotherapy in HCC, the integration of m6A modulators with conventional therapeutic modalities is anticipated to represent an effective strategy to overcome drug resistance and improve treatment outcomes.
